# Supplementary figures and images for: Design of a new multiplex PCR assay for rice pathogenic bacteria detection and its application to infer disease incidence and detect co-infection in rice fields in Burkina Faso
Source: PLoS One. 2020 Apr 27;15(4):e0232115. doi: 10.1371/journal.pone.0232115 (PMC7185701; doi:10.1371/journal.pone.0232115)

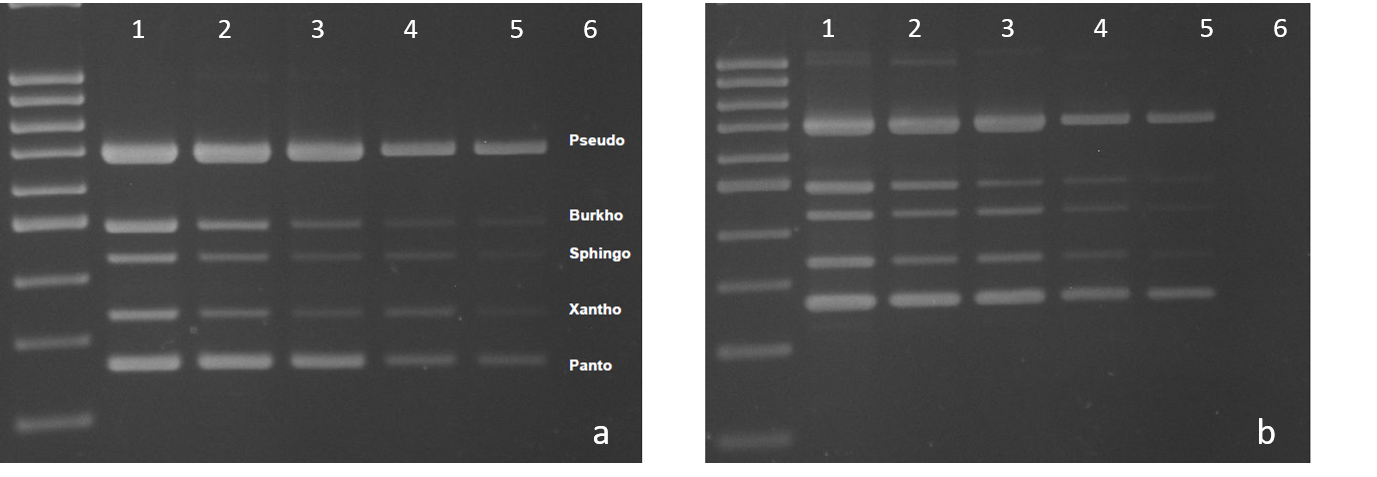

Supplement: S1 Fig — a: multiplex PCR with (NH4)2SO4; b: multiplex PCR without (NH4)2SO4. Every reaction was performed with a mix of six samples of each control bacterial strain (Pseudomonas fuscovaginae strain UBP735, Burkholderia glumae strain NCPPB 3923, Sphingomonas spp. strain V1-2, Xanthomonas oryzae pv. oryzae strain BAI10, Pantoea ssp. strain ARC10) at different concentrations. Lane 1: 5 ng/μl, lane 2: 1 ng/μl, lane 3: 0.5 ng/μl, lane 4: 0.1 ng/μl and lane 5: 0.05ng/μl, lane 6: water control. (TIF) [file pone.0232115.s003.tif]

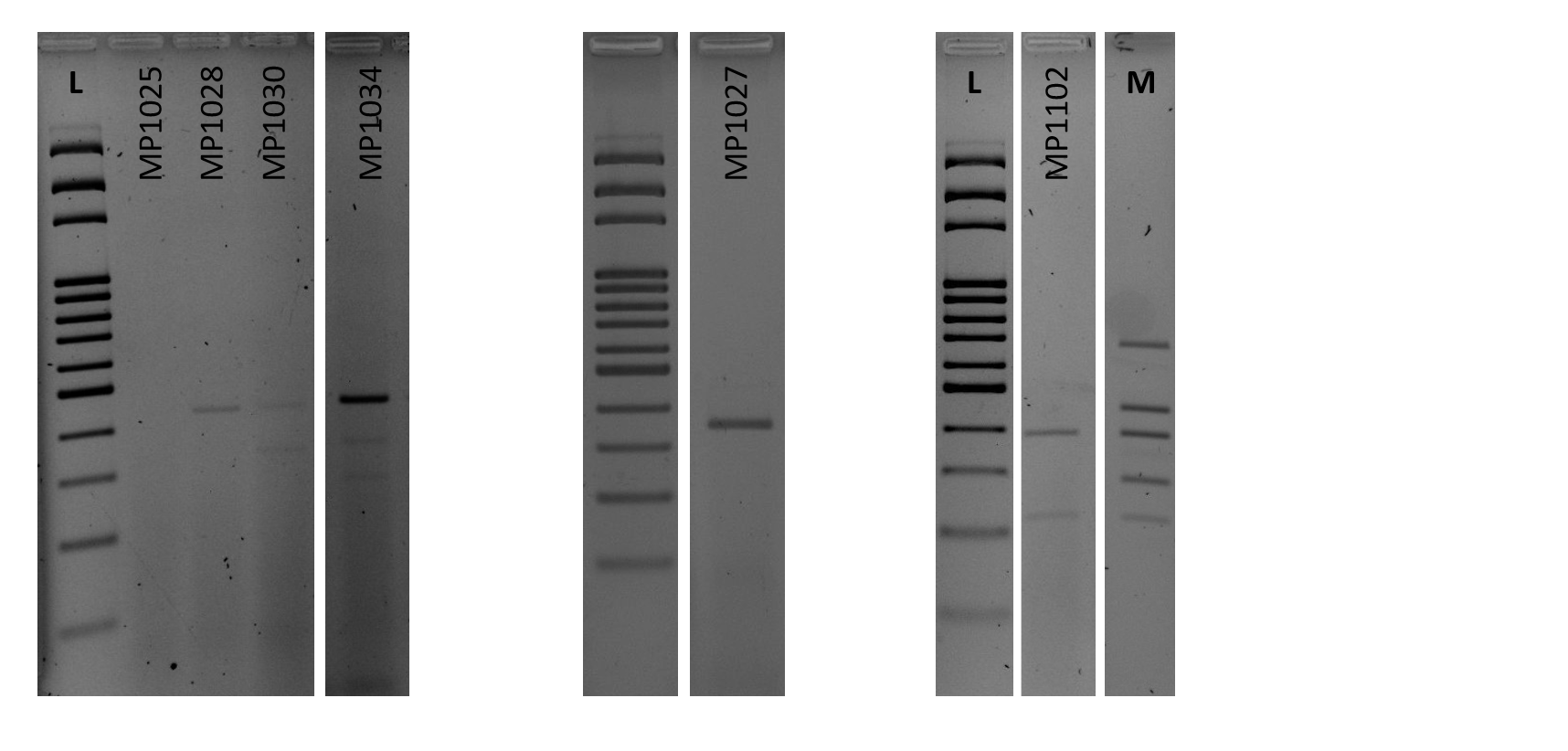

Supplement: S2 Fig — Example of detection of different bacterial taxa from field samples. Six samples were chosen to present the different possibilities obtained. L: molecular size marker, 100pb DNA ladder ready to load, Solis Biodyne. M: all five bacterial DNA samples. (TIF) [file pone.0232115.s004.tif]

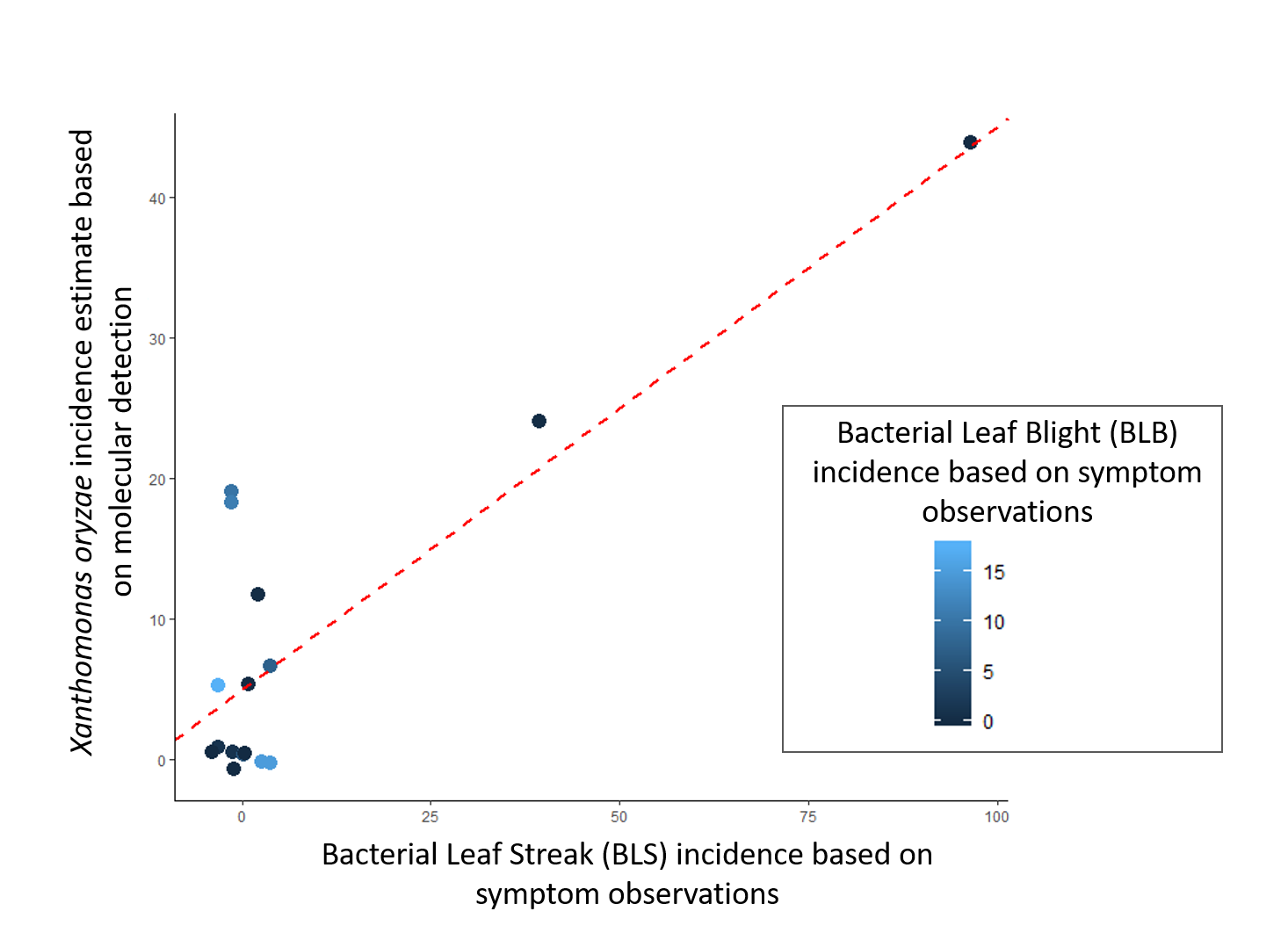

Supplement: S3 Fig — Each panel corresponds to one of the targeted bacterial taxa. For each of the two studied irrigated areas (Sites: Banzon and Karfiguela), both the boxplot, as well as the points corresponding to incidence estimates for each field, are given. (TIF) [file pone.0232115.s005.tif]

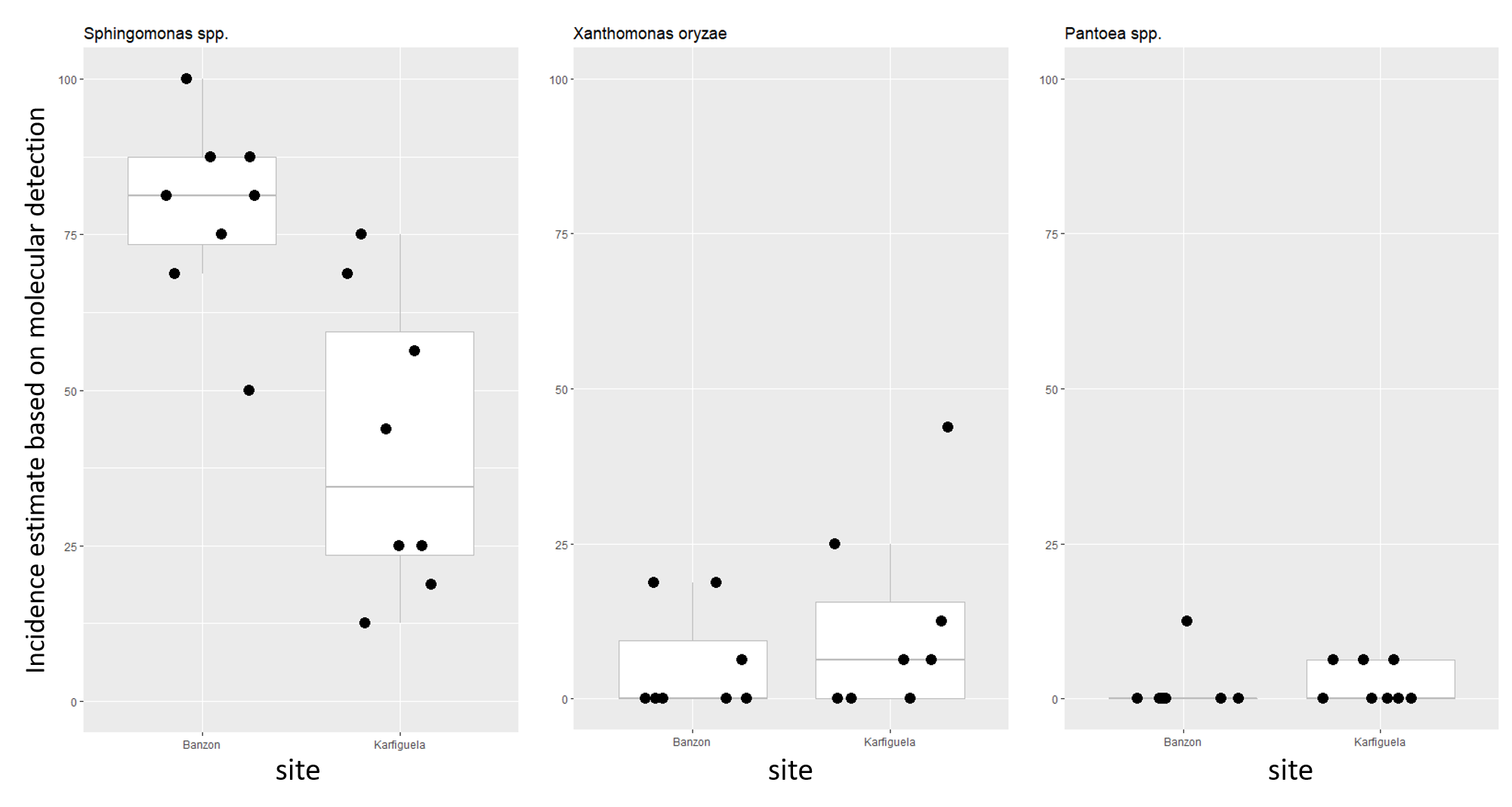

Supplement: S4 Fig — Each point corresponds to one field, with BLS incidence estimate based on symptom observations on the x-axis and Xo incidence based on molecular detection on the y-axis (the red dotted line representing the linear regression between the two variables). Color of the points reflects BLB incidence estimate based on symptom observations. (TIF) [file pone.0232115.s006.tif]

**Figure 1**

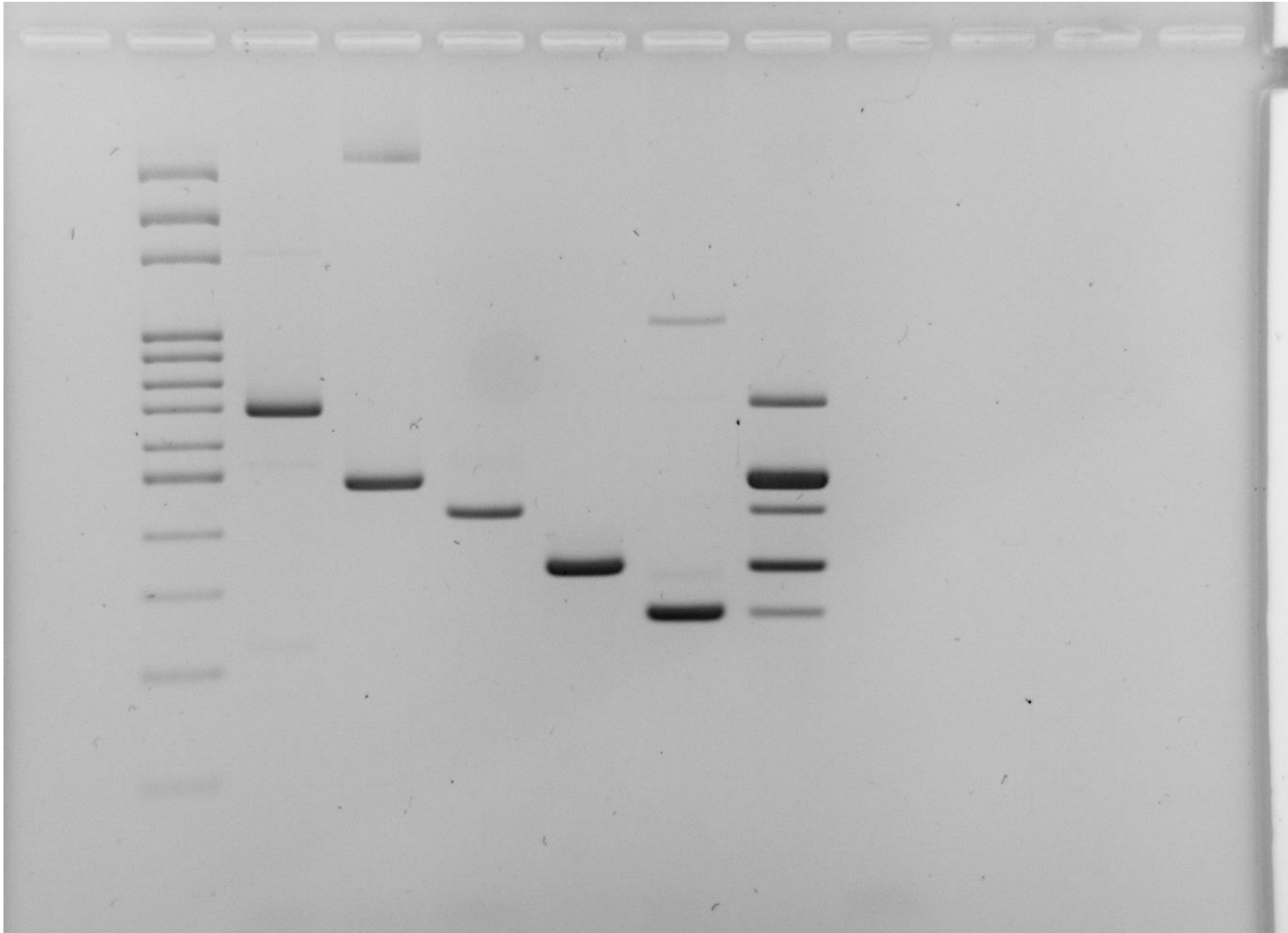

**Figure 2**

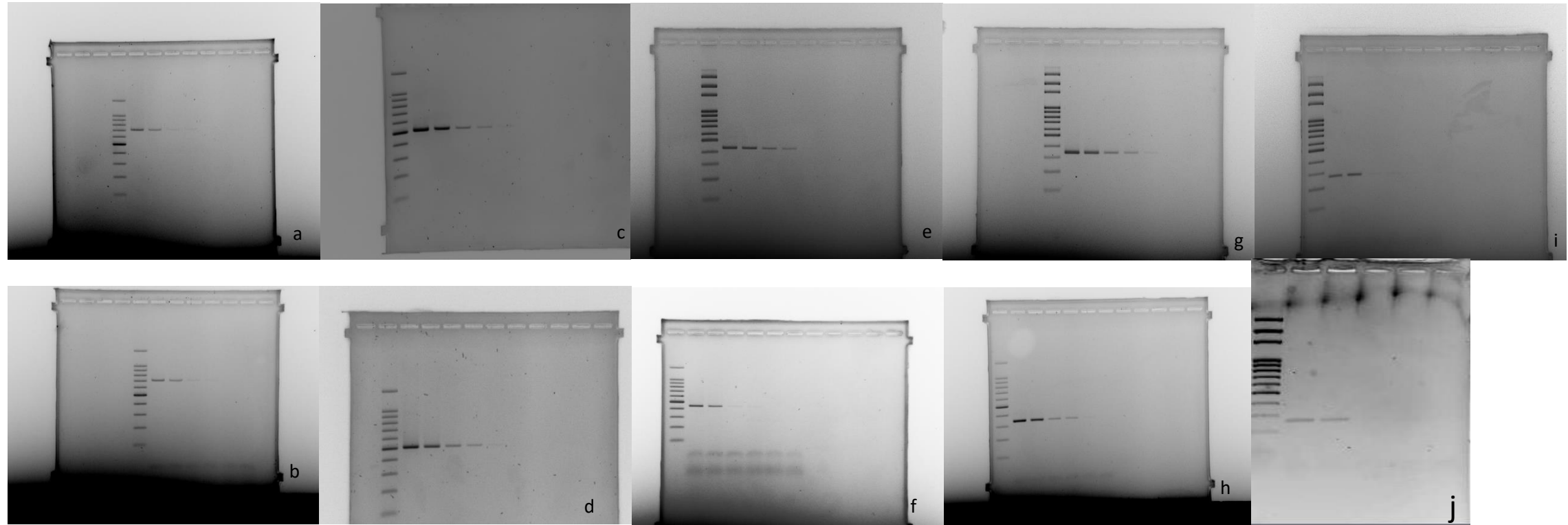

**S1 Figure**

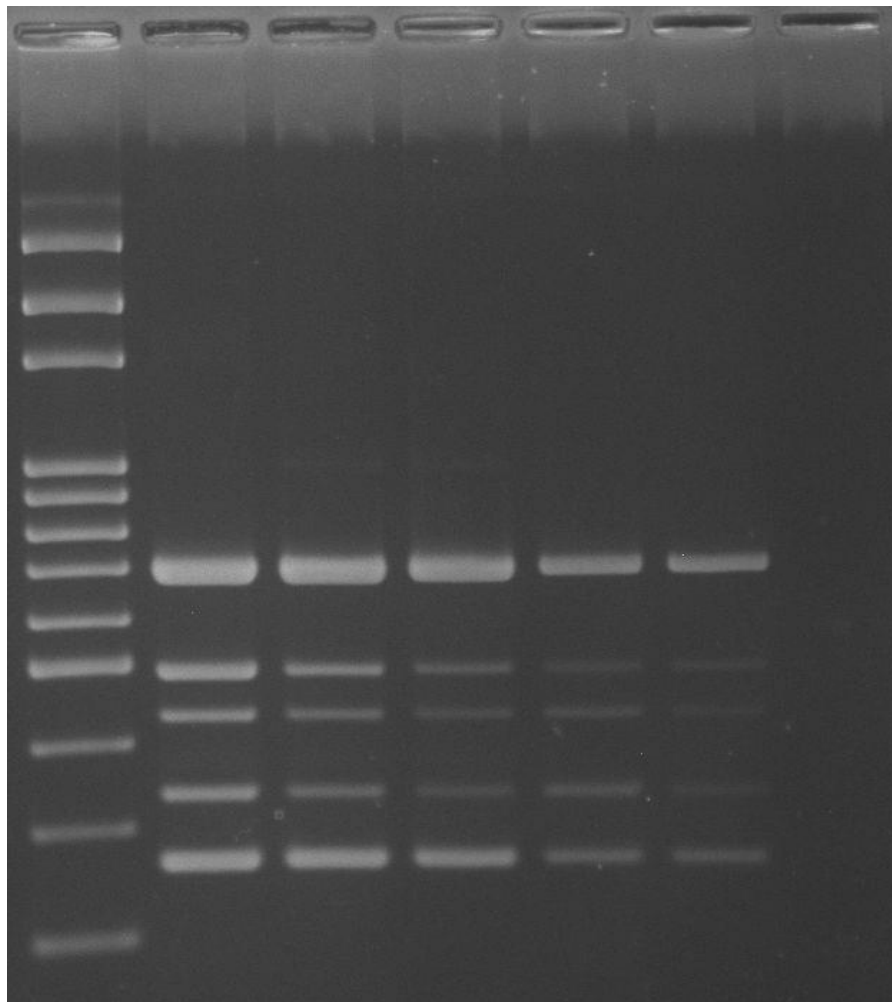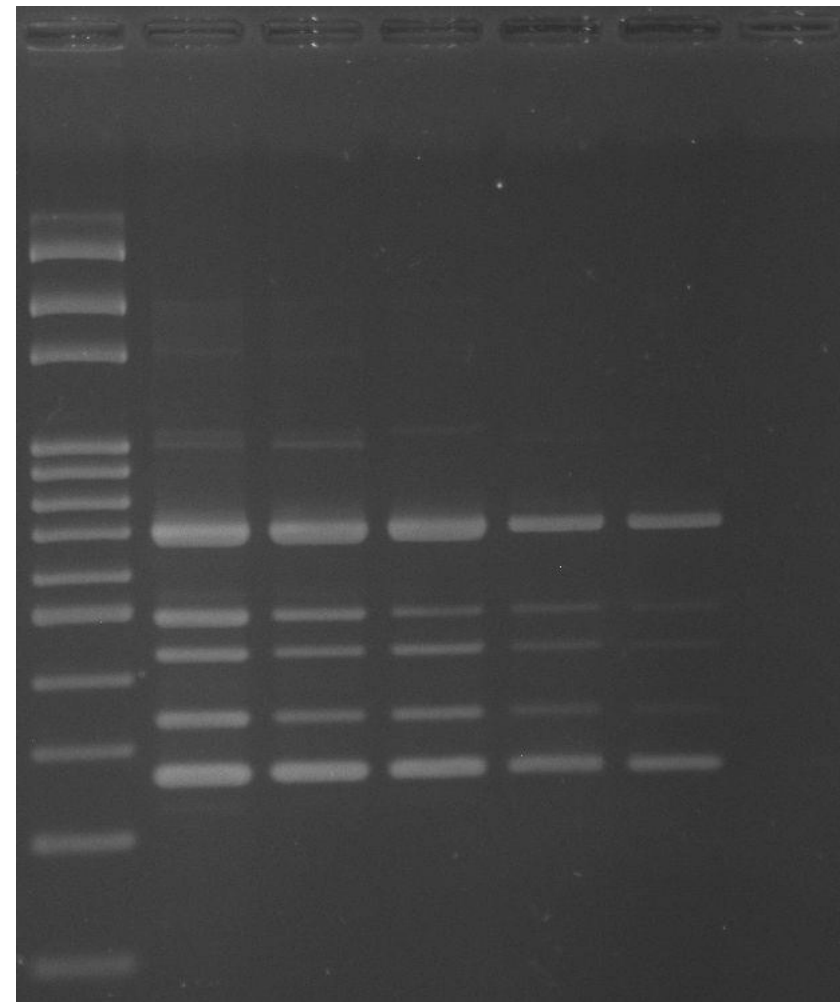

**S2 Figure**

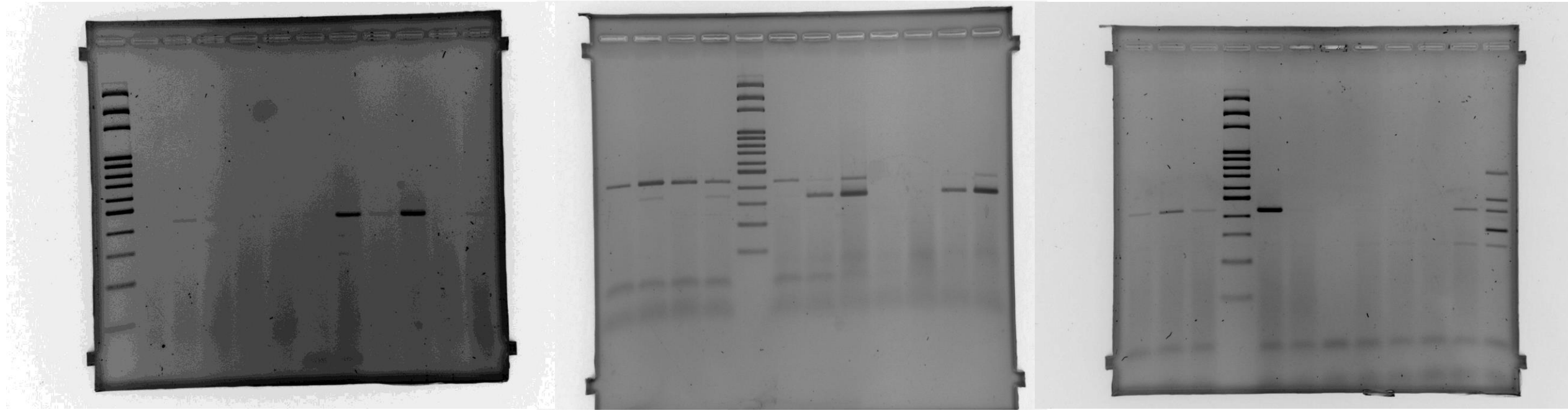

Supplement: S1 Raw images — (PDF) [file pone.0232115.s007.pdf]
