# Supplementary material for: Design of a new multiplex PCR assay for rice pathogenic bacteria detection and its application to infer disease incidence and detect co-infection in rice fields in Burkina Faso
Source: PLoS One. 2020 Apr 27;15(4):e0232115. doi: 10.1371/journal.pone.0232115 (PMC7185701; doi:10.1371/journal.pone.0232115)
Supplement: Attachment — Submitted filename: Reviewer Comments.docx [file pone.0232115.s008.docx]

**Reviewer Comments:**

The manuscript entitled “Design of a new multiplex PCR assay for rice pathogenic bacteria detection and its application to infer disease incidence and detect co-infection in rice fields in Burkina Faso” by Bangratz M. et al., indicate multiplexing technique used for identification of bacterial pathogen in the rice field. The authors have identified unique refence primers for identification of five texa of plant pathogens viz. *Pseudomonas, Xanthomonas, Burkholderia, Sphingomonas,* and *Pantoea* species. The authors standardized PCR condition for multiplexing and used the method to identify bacterial infestation in rice field at Burkina Faso. However, there are some severe concerns about the manuscript that need to be addressed for manuscript to meet applicable standards for publication.

Major comments:

1. The authors claim that infestation of Sphingomonas or Pantoea spp. leads to bacterial leaf blight (BLB) like phenotype. However, there are not clear evidences to claim it. Please provide some images of leaves showing such BLB like phenotype. It will be good if authors can provide images of phenotype and indicate in which leaf which bacteria/s were observed. *Xanthomonas* *oryzae* pv. *oryzae* is known to cause BLB symptom (disease lesion in the mid-vein). Did the authors perform leaf-clip inoculation experiment and seen the similar BLB like phenotype with Sphingomonas or Pantoea spp.?
2. The multiplex PCR is the major finding of this manuscript. However, there is not even a single gel image indicating to diagnose this in the field/infected plants. A figure (gel picture) in main text must be added where template genomic DNA is from the infected plant samples (This should be shown for at least KA02 and KA09: where 3 pathogens have been detected).
3. Authors detected only 3 pathogens in the field samples. Is it because of sensitivity of the assay or absence of the pathogen?

Authors should infect plants with the pathogen individually, pool the infected parts, isolate genomic DNA and perform the multiplex PCR.

4. Line 32-33 and 261-262 and Table 3 data: The data in Table 3 indicates the BLB phenotype is majorly because of *Sphingomonas* spp. As most of the fields showing BLB phenotype are negative for *Xanthomonas* in molecular diagnostic (Table 3). In the abstract (lines 32-33) and discussion (Lines 261-262) it is reported that “Xanthomonas oryzae incidence levels were in congruence with bacterial leaf streak (BLS) and bacterial leaf blight (BLB) symptom observations in the field” .
5. In Table 3, BLB and BLS column values (such as 17, 95 and 40) are not clear. If 16 plants were observed in the field, what these numbers represent?
6. Reduce size of introduction. Precisely reduce the part where details of all diseases is provided (line 61-96). In the last paragraph of introduction, add little details of findings of this manuscript.
7. Why there is such a huge variation in primer concentration used for different species?

Minor comments

1. Line 230; mention ‘42 strains of five bacterial texa’.
